# Temperature Measurements in Rehabilitation in Patients with Completely Ruptured Anterior Cruciate Ligament before and after RegentK and Physiotherapy

**DOI:** 10.3390/medicines1010012

**Published:** 2014-07-16

**Authors:** Gerhard Litscher, Daniela Litscher, Michael Ofner, Ingrid Gaischek, Daniela-Eugenia Malliga

**Affiliations:** 1 Research Unit for Complementary and Integrative Laser Medicine, Medical University of Graz, Auenbruggerplatz 29, 8036 Graz, Austria; E-Mails: daniela.litscher@medunigraz.at (D.L.); ingrid.gaischek@medunigraz.at (I.G.); 2 Research Unit of Biomedical Engineering in Anesthesia and Intensive Care Medicine, Medical University of Graz, Auenbruggerplatz 29, 8036 Graz, Austria; 3 TCM Research Center Graz, Medical University of Graz, Auenbruggerplatz 29, 8036 Graz, Austria; 4 Department of Sports Physiology, University of Vienna, Auf der Schmelz 6, 1150 Vienna, Austria; E-Mail: michael.ofner@medyco.net; 5 Division of Cardiac Surgery, Department of Surgery, Medical University of Graz, Auenbruggerplatz 29, 8036 Graz, Austria; E-Mail: daniela-eugenia.martin@medunigraz.at

**Keywords:** RegentK, Khalifa therapy, physiotherapy, anterior cruciate ligament, skin temperature, thermal imaging

## Abstract

Acute skin surface temperature effects on the knee were investigated after a manual therapy developed by Mohamed Khalifa (RegentK) compared to standard physiotherapy in patients with completely ruptured anterior cruciate ligament (ACL). Twenty patients participated in this study. They were randomly assigned to group A (receiving RegentK) or group B (physiotherapy). Each group consisted of 10 patients. Temperature values were registered on four spots (three on the knee, one on the foot) of the injured and the healthy leg (control). Skin temperature increased significantly after RegentK on all sites of the injured leg, but after physiotherapy only the measurement spots on the knee showed significant increases. After RegentK the temperature had also increased significantly on the control leg, whereas in group B, the results were not significant. Experimental and clinical testing of technical equipment, e.g., infrared thermography, for ACL injuries is important for a better understanding of the different physiological/pathophysiological mechanisms underlying different therapy approaches.

## 1. Introduction

According to Statistics Austria [1], about 630,000 Austrians get hurt in their spare time and when practicing sports. This is followed on the one hand by short-, medium-, and sometimes also long-term restrictions in their quality of life, connected with pain and limited mobility of joints, and on the other hand huge health-economical costs for diagnosis, acute therapy and rehabilitation. Every objective idea for improvement and every possibility with the potential to have preventive effects and/or be more effective in rehabilitation than current measures should therefore be discussed as an option to standard therapies. If, for example, a non-invasive treatment form was an efficient alternative to an arthroscopy, this would be a huge step forward in medicine, bringing about enormous potential to save time and costs. This might also reduce the occurrence rate of side effects and drastically increase the patients’ quality of life.

One of these new therapy methods is RegentK, a non-invasive pressure therapy which was developed and practiced by Mohamed Khalifa in Hallein, Austria [2,3,4,5,6]. With this new technique, it is possible to accelerate the healing process of totally ruptured anterior cruciate ligaments (ACL) [6].

Quantitative thermal imaging is becoming an important method in rehabilitation research. Temperature measurements are justified because of their non-invasiveness and the possibility to perform these measurements without skin contact. Using infrared thermography, we evaluated the amount of changes in skin surface temperature on the knee and periphery before and after RegentK to a standard physiotherapy within a randomized controlled study.

## 2. Materials and Methods

### 2.1. Patients

In this study, we investigated 20 patients who were randomly assigned to the two intervention groups (group A: RegentK; group B: physiotherapy). Each group consisted of 10 patients. In group A, patients had a mean age ± SD of 31.3 ± 8.5 years (range 20–43 years). Their mean height was 170.5 ± 10.4 cm, and their mean body weight was 67.7 ± 12.2 kg. Eight were female and two male. The injury had been caused by skiing (*n* = 8), household activities (*n* = 1) and volleyball (*n* = 1). Five patients had a ruptured ACL in the right knee, five in the left. Patients in group B were 34.8 ± 10.2 years old (range 19–47 years), had a height of 174.8 ± 9.7 cm and a weight of 66.1 ± 12.0 kg. Six were female and four male. Again, skiing was the main cause of the injury (*n* = 9), volleyball (*n* = 1) the other. The ruptured ACL was in the left knee of four patients and in the right knee of the remaining six patients.

Inclusion criteria were: (a) unilateral complete rupture of the ACL (less than 14 days ago), verified by magnetic resonance imaging; (b) age: 18–49 years; (c) normal body weight: BMI (body mass index) 18–25; (d) regular exercise level; (e) knee instability: experienced at least one giving-way; and (f) dysfunction: knee range-of-motion: reduced or inhibited.

Exclusion criteria were: (a) preceding surgical intervention (including arthroscopy) on the injured knee; (b) metabolic disorders like diabetes mellitus; and (c) autoimmune diseases.

The study was approved by the ethics committee of the University of Salzburg, Austria (21-232 11-12 sbg + amendment), and registered at clinicaltrials.gov under the ID-No. NCT01762371. The patients were informed about the nature of the investigation as far as the study design allowed and provided written informed consent.

### 2.2. RegentK

RegentK, the manual therapy developed by Mohamed Khalifa, is described in detail in a previous paper [4], “Khalifa therapy is described as functional-pathological [2]. In this approach, function is the primary concern, not anatomy. The most important thing is not the ruptured ligament itself, but its function/dysfunction. Khalifa therapy restores the function of the knee in a natural way. During the 60–90 min of his manual therapy, he applies pressure to the injured knee in order to activate the self-healing processes of the human body, using his hands as an instrument for both measurement and therapy. Over periods of varying length, he applies increasing pressure on a spot before moving on to the next spot. The frequency of pressure application depends on the patient’s physiological reaction. The force of the pressure is not comparable to that normally used in acupressure in Traditional Chinese Medicine [7]; it is much higher. Mohamed Khalifa’s method is based on manual pressure of varying frequency and does not damage the body, but supports it in its own natural healing activities. If one cuts through an elastic band and sews it together again, one cannot expect it to be as elastic at the stitching point as it was before. It is the same with human ligaments, and if the elasticity is disrupted anywhere in the human body, the whole system is affected [2,3]”. RegentK consists of only one session.

### 2.3. Physiotherapy

Different myofascial treatment methods were applied during physiotherapeutic intervention in order to mobilize/activate the knee joint. Focus was put on the myofascial structures of tissues surrounding the knee joint; the upper part of the body and the upper extremities were not included in the treatment. Two basic techniques were used during the control intervention: (a) a static manual treatment, *i.e.*, pressing trigger points usually to be found in the muscle belly (typically e.g., in the m. quadriceps, the m. biceps femoris, m. popliteus, m. gastrocnemius); and (b) static-dynamic stretching intermuscular techniques in the areas of the tractus iliotibialis, the medial femoral muscles directed toward the knee or between (and in) the muscle bellies of the m. gastrocnemius. Both techniques were performed at the patient’s individual pain threshold, and static trigger points were pressed 3–5 times (for a maximum of 30 s each) until the tension and pain subsided. The myofascial longitudinal and crosswise stretchings were also repeated 3–5 times, slowly and dynamically. Before and during the treatment (duration about 60 min), patients were informed about potential physiologic reactions of the interventions [8,9,10,11,12]. In this study, physiotherapy also consisted of only one session.

### 2.4. Technical Equipment for Thermal Imaging

The temperature measurements were performed using a Flir i7 (Flir Systems, Wilsonville, OR, USA) infrared camera which operates at a wavelength range from 7.5 to 13 µm. The focal distance of the IR lens is *f* = 6.8 mm. The temperature measurement range lies between −20 °C and +250 °C. Its accuracy lies at ±2% of the reading. Sensitivity is <0.1 °C at 30 °C, and the infrared resolution is 140 × 140 pixel. The system is ready for use in 15–20 s.

### 2.5. Procedure

The thermal imaging measurement procedure was the same for both patient groups. Approximately 3 h before starting the measurement, both legs were shaved. Thermal images were taken before the start and after the end of the respective manual therapy.

All patients were investigated in a supine position under similar conditions. Immediately before and immediately after therapy, six thermographical recordings were performed, respectively: in lateral and medial position of both the injured and the healthy (control) knee (which did not receive any therapy or manual manipulation), and one of each entire leg. Pictures were analyzed using the software provided with the camera. The temperature was evaluated at three different spots on the knee: anterolateral, frontal, and anteromedial. The measurement spot for assessing the temperature changes on the foot was located at the great toe (left and right, respectively).

Environmental temperature was also recorded before and after the treatment.

### 2.6. Statistical Analysis

The temperature values of both legs were tested with paired *t*-test (before *vs.* after; SigmaPlot 12.0, Systat Software Inc., Chicago, IL, USA). The level of significance was defined as *p* < 0.05.

## 3. Results and Discussion

All 20 patients completed the study procedure, and the thermographical measurements could be carried out without problems. The demographical data showed no significant differences between RegentK group and physiotherapy group.

**Figure 1:**
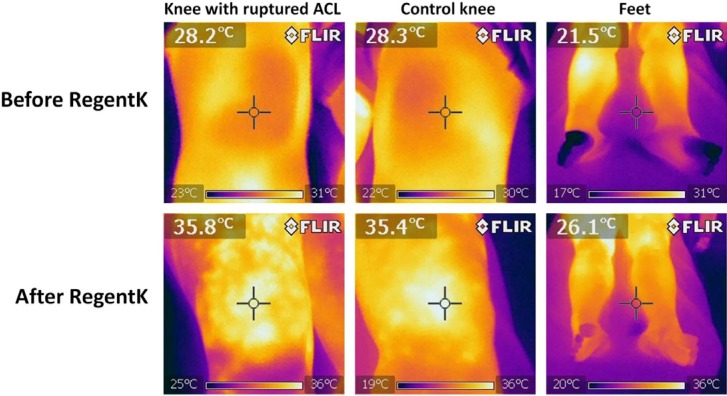
Thermal imaging in one patient before and after RegentK. Note the increased temperature at the injured and also the control knee after treatment. It can also be seen that the temperature is initially higher on the injured knee.

Figure 1 shows a typical example of a patient before and after RegentK.

**Figure 2:**
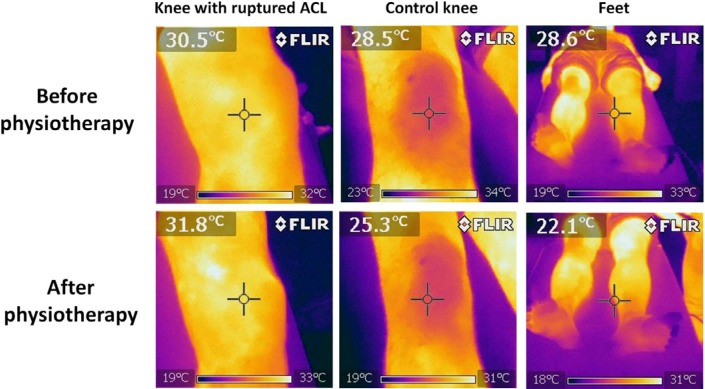
Thermal imaging performed in a patient before and after physiotherapy. The effects are similar to those after RegentK; however, the increase in temperature is not as high as after RegentK.

Figure 2 shows six thermal images from a patient before and after physiotherapy.

**Figure 3:**
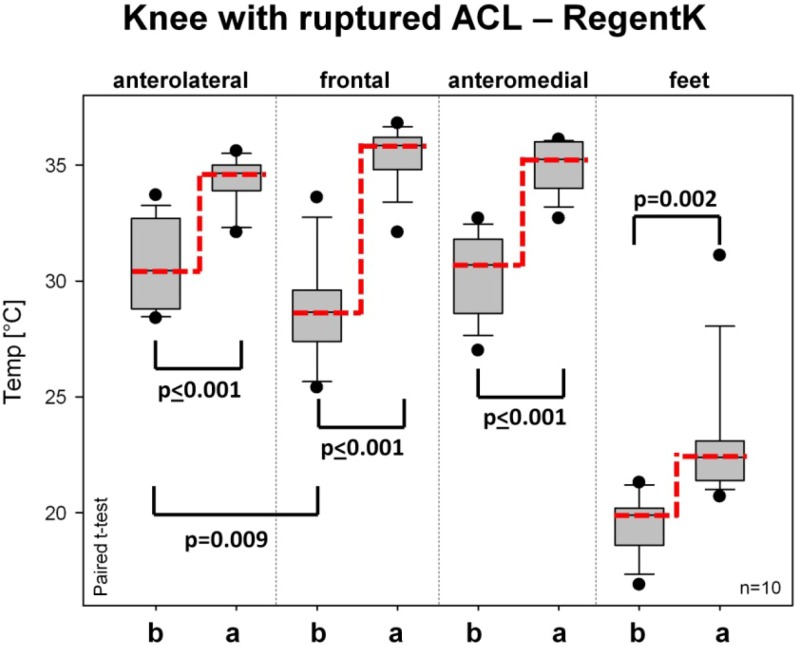
Box plot presentation of changes in skin surface temperature on the injured knee after RegentK (ends of boxes: 25th and 75th percentile; line at the median; error bars: 10th and 90th percentile).

A highly significant (*p* ≤ 0.001) temperature increase after RegentK can be seen on all measurement spots of the injured knee in Figure 3. The measurement spot on the foot also showed a significant increase.

**Figure 4:**
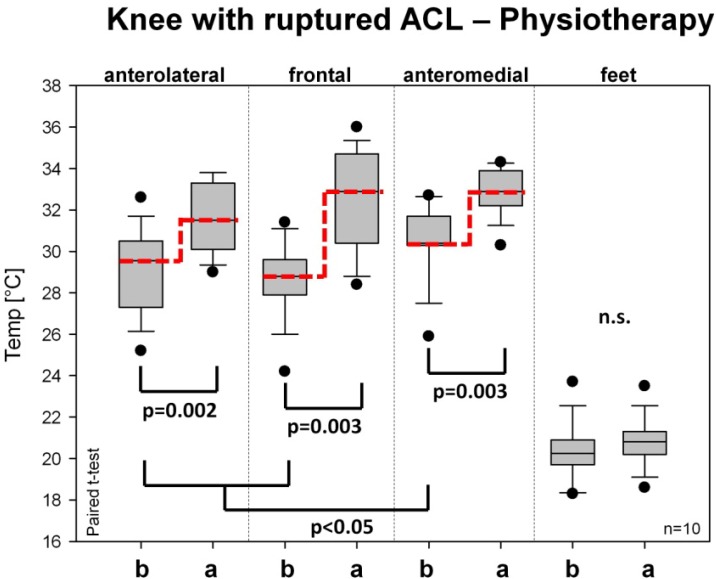
Changes in skin temperature on the injured knee before and after physiotherapy. Further explanations see Figure 3.

Figure 4 shows the temperature values on the injured knee before and after physiotherapy. The baseline values before treatment were similar to those in group A. However, the increase after physiotherapy was far less than after RegentK. The level of statistical significance was reached on all measurement spots on the knee, but not on the foot.

**Figure 5:**
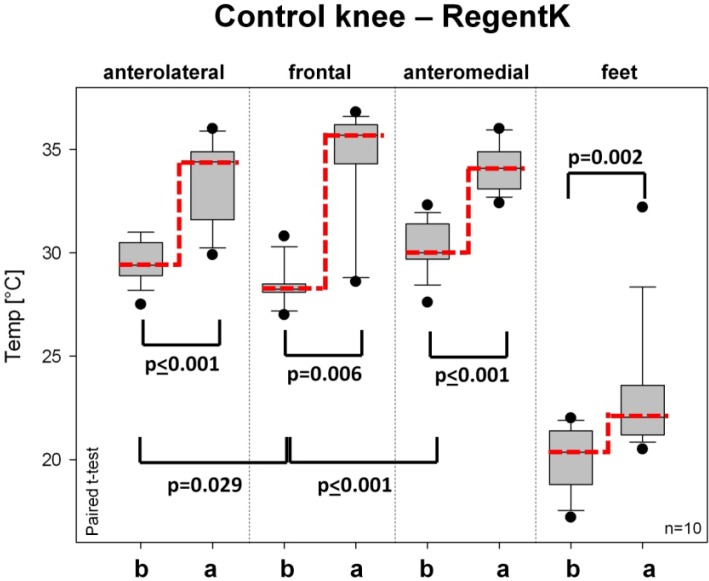
Temperature changes on the healthy control knee before and after RegentK. For further explanations see Figure 3.

**Figure 6:**
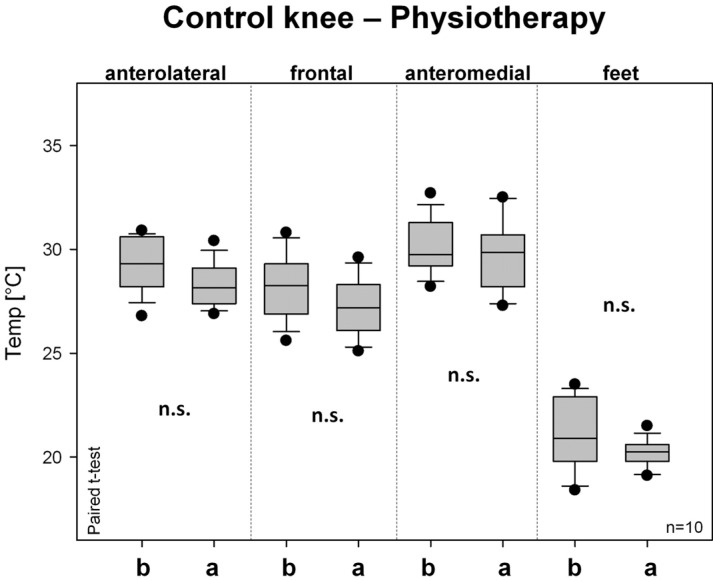
Changes in skin surface temperature on the healthy control knee before and after physiotherapy. For further explanations, see Figure 3.

Figure 5 and Figure 6 show the skin surface temperature on the control (healthy) knee. The baseline values before therapy were similar to or slightly lower than those of the injured knee. On all measurement sites of the healthy knee, there was also a significant increase after RegentK (Figure 5).

Figure 6 summarizes the temperature changes of the control knee before and after physiotherapy. No significant changes were found in any of the evaluated sites.

Room temperature showed no significant differences before and after RegentK (21.8 ± 1.0 °C before; 22.4 ± 1.1 °C after) or before (20.5 ± 0.4 °C) and after (20.4 ± 0.2 °C) physiotherapy. Since the therapies took place in different buildings, there were differences in room temperature between the two locations, before as well as after the respective kind of therapy.

The function of all 10 knees with a ruptured ACL was restored after RegentK (treatment duration (mean ± SD): 77.8 ± 10.6 min), but not after 57.2 ± 9.4 min of physiotherapy. All patients in the RegentK group were able to bend their knees, to jump, to run on the spot and to perform squats with only the injured leg immediately after the end of the treatment. In the physiotherapy group, some success could also be seen, but not to the same extent as in group A. The clinical data will be presented in detail by other research teams.

The opinion exists that the first phase of physiotherapy after ACL injury should be focused on anti-inflammatory (*i.e.*, reducing the joint temperature), analgesic, anti-oedemic procedures and procedures that prevent muscle atrophy. That means that procedures increasing the temperature of the involved knee joint would be quite opposite to the aim of the first phase of physiotherapy after ACL rupture. However, our study could not confirm this hypothesis, at least for RegentK, although it should be noticed that we only measured surface temperature and not the temperature in the depth.

Infrared energy lies within a wavelength range no longer detectable by the human eye, in the region of the electro-magnetic spectrum which is perceived as being warm. Contrary to visible light, every object in this region with a temperature over absolute zero radiates heat. When the temperature of an object is higher, the intensity of emitted infrared radiation is also higher. Infrared cameras produce pictures of invisible infrared- or heat radiation, thus enable exact temperature measurements [13].

The technique of infrared temperature measurement has already been used in several studies dealing with massage or physiotherapy [14,15,16]. Advantages of this method are the high local resolution as well as optical data registration without requiring skin contact. In addition, the method is passive, *i.e.*, no energy is transmitted into the body and thus is completely harmless. This allows longer examination times.

The results of thermal images after manipulative techniques are still discussed controversially in scientific literature. Some papers describe cooling effects after osteopathic manipulative treatment [15], whereas others like our results showed massive increases in temperature after physiotherapy and RegentK. The latter effects of RegentK were already described in a previous study [5]. The results of the current study are directly comparable to the first preliminary results obtained about a year ago. In both studies, highly significant increases of temperature were seen on the injured knee after RegentK. The current results are new and interesting because they compare RegentK with a standard therapy (physiotherapy) for the first time. While after RegentK, temperature had increased significantly also on the healthy, non-treated knee, which was not the case for physiotherapy. Thus it can be concluded that the mechanisms underlying the two therapies are different. Physiotherapy showed only local temperature effects; RegentK, in contrast, led to local effects, but also to effects which are potentially related to the neurovegetative system. Therefore we also recorded electrodermal activity using a new system developed by our research team, and we will describe this in another article.

Accurate diagnosis of ACL injuries relies on injury history [17], clinical assessment [18], as well as advanced imaging techniques [19]. In scientific literature, “imaging techniques” mostly refers to magnetic resonance imaging. It is interesting that in our investigation we found that temperature in the area of the rupture was higher than on the control knee, but that the temperature in the periphery of the injured leg was lower than on the healthy leg. 

## 4. Conclusions

In conclusion, experimental and clinical testing of technical equipment in rehabilitation medicine, especially for ACL injuries, is important for a better understanding of the underlying physiological and pathophysiological mechanisms. Due to improvements in thermography within the last years, infrared cameras can be used without any technical problems. Maybe this simple technical method of thermography can be integrated in future computer-assisted biomechanical video systems. In our study we were able to demonstrate the effects of RegentK in comparison to a standard method of physiotherapy using modern infrared thermography. The results clearly demonstrate that RegentK influences skin surface temperature significantly more than physiotherapy.

Thermographic methods such as infrared cameras at different wavelengths (ranges of 2–13 µm) are effective methods in evidence-based research which support demystification of complementary treatment methods. However, the validity of the method must be considered critically and analyzed scientifically. The method is dependent upon many physical-technical aspects, e.g., thermic reflections [13]. Therefore, one should be very careful in analyzing thermographic images.

As a future aspect, a thermographic video system may record entire treatment sessions (e.g., RegentK and physiotherapy) to visualize the changes in temperature with a higher temporal resolution.
